# Finite element analysis of electric field distribution during direct current stimulation of the spinal cord: Implications for device design

**DOI:** 10.1063/5.0163264

**Published:** 2023-11-02

**Authors:** Joe G. Troughton, Yaw O. Ansong Snr, Nida Duobaite, Christopher M. Proctor

**Affiliations:** 1 Department of Engineering, University of Cambridge, Trumpington Street, Cambridge, United Kingdom; 2 Department of Engineering Science, Institute of Biomedical Engineering, University of Oxford, Oxford, United Kingdom

## Abstract

Spinal cord injury (SCI) arises from damage to the spinal cord, often caused by trauma or disease. The resulting sensorimotor dysfunction is variable and dependent on the extent of the injury. Despite years of research, curative options for SCI remain limited. However, recent advancements in electric field stimulated axonal regrowth have shown promise for neuronal regeneration. One roadblock in the development of therapeutic treatments based on this is a lack of understanding of the exogenous electric field distribution in the injured tissue, and in particular, how this is influenced by electrode geometry and placement. To better understand this electric field, and provide a means by which it can be optimized, we have developed a finite element model of such spinal cord treatment. We investigate the impact of variations in electrode geometry, spinal cord size, and applied current magnitude as well as looking at several injury models in relation to clinically observed outcomes. Through this, we show that electrode shape has little effect on the induced electric field, that the placement of these electrodes has a noticeable influence on the field distribution, and that the magnitude of this field is governed by both the applied current and the spinal cord morphology. We also show that the injury modality influences the induced field distribution and that a stronger understanding of the injury will help decide treatment parameters. This work provides guidance in the design of electrodes for future clinical application in direct current electric field stimulation for axonal regeneration.

## I. INTRODUCTION

Spinal cord injury (SCI) occurs when there is an insult that disrupts the neuronal tissues within the spinal cord. This can be due to a disease process, degeneration, or trauma.^1,2^ Damage to the neuronal cells following SCI leads to the clinical features of sensorimotor dysfunction. The extent and location of this damage, which can manifest as either complete or incomplete injury, play a crucial role in determining the range of sequelae that follow SCI.^3,4^

According to the World Health Organization, up to half a million new cases of spinal cord injuries occur globally per annum.^5^ Despite the existence of numerous promising interventions, spinal cord injury remains a challenging condition to treat, with mortality rate as high as 36.5% in the first year post injury.^6^ Yet therapeutic options remain limited, and a cure following SCI is yet to be achieved.^2,7,8^ In this context, the urgent need for effective therapies that can improve the prognosis and quality of life for individuals with SCI is paramount.

Extensive scientific research has demonstrated the effect of an exogenous electric field on neuronal regeneration. By acting as neurotrophic and neurotropic enablers, supporting the growth and ordering of neural tissues, these electrical currents provide the crucial elements for neuronal regeneration.^9–15^ This opens up new possibilities for the development of novel therapeutics aimed at functional recovery following SCI. A broad range of electric field magnitudes has been reported as effective for neuronal regeneration in various studies. Notably, Ingvar^16^ demonstrated the effectiveness of an electric field intensity of 0.01 mV/mm, while Marsh *et al.*^17^ observed promising results at 50 mV/mm. Hinkle *et al.*^18^ further expanded the spectrum, reporting a range of 7–190 mV/mm, while Jaffe and Poo^19^ found the range of 70–140 mV/mm to be conducive to neuronal regeneration. These reports, all based on *in vitro* studies of neuronal tissue cultures, provide strong evidence for the potential efficacy of electric field based treatments. However, they lack coherence as a body of work due to the variety of donor organisms and protocols used for the cell culture preparation and treatment, making direct comparison between reports challenging. Nonetheless, there is a growing consensus that neuronal growth increases toward the cathode almost immediately^9,14,18^ with increased filopodial sprouting on cathodal facing neurites,^20,21^ while anodal facing neurons slow down,^9,19,21^ retract,^14^ resorb,^21^ or reverse.^17^ Since the initiation of cathodal growth of the axons outpaces axonal retraction and/or resorption at the anode, reversing the electric field polarity within this biological window can result in bidirectional axonal growth across the lesion site. This periodic reversal of the field gives rise to the common term oscillating field stimulation (OFS) and has a time window estimated to be in the region of minutes to hours,^21^ with 15 min commonly used as the period in clinical applications.^11,13,15,22^ This is an evolution of the initial stimulation regime of a fixed polarity, or direct current (DC) stimulation, which dominated early research in electrical stimulation of axonal regrowth. While OFS can be considered a form of alternating current (AC) stimulation, the very low frequencies (∼1 mHz) mean it is appropriate to consider OFS as a distinct stimulation paradigm, with features of both AC and DC stimulation. As with AC stimulation, OFS has some concern for the amount of charge delivered before the polarity is reversed, known as the charge per pulse. However, because of the long time spans between polarity reversal, the electrical behavior of the stimulated system is more akin to that of DC stimulation. An added advantage of this polarity reversal is that it neutralizes the accumulated neurotoxic electrochemical products from reversable Faradic reactions,^11,23^ including metal oxides on the electrode surface, changes in pH near the electrode surface,^24^ and to a lesser extent the products of water electrolysis.^25^ However, products from irreversible Faradic reactions, including the majority of electrolysis products, as well as those from the oxidization of saline and organic species,^26^ are not removed, and therefore should be avoided where possible.

Although clinical trials in the past have shown promising results, the translation of OFS technology has encountered significant impediments over the last decade, primarily attributable to the unavailability of an appropriate implantable platform for delivering safe yet effective electric fields to spinal cord tissue over prolonged periods. Previous attempts at OFS intervention have relied on bulky platforms,^11,15^ and typically utilized conventional electrodes composed of platinum^11,15^ and iridium.^27^ This poses several drawbacks. First, the bulky nature necessitates multiple surgeries for implantation and explantation, impacting on patient morbidity. Additionally, OFS therapy requires the injection of large amounts of charge per pulse. In conventional bare metal electrodes, the charge injection capacity (CIC) is limited owing to the small double layer capacitance of such materials, on the order of a few mC/cm^2^,^28^ leading to toxic irreversible Faradaic reactions necessary to facilitate continued change injection after approximately one second.^11,13^ More recent work has sought to increase the CIC of metal electrodes, predominantly by increasing the surface area of the electrode through surface structuring induced during,^29–34^ or post, deposition.^28^ While these strategies have increased the CIC values by up to an order of magnitude, they are still far short of the values required for OFS therapies. On the other hand, recent research has shown that high capacitance coatings with conducting polymers that exhibit volumetric capacitance can enhance the capacitance of bare metals of the same area by more than 100-fold.^35–38^ Thick films of such high capacitive materials may allow for direct current stimulation for tens of minutes without irreversible Faradaic reactions. However, it should be noted that recent studies have shown that, under certain stimulation conditions, conducting polymers still lead to irreversible Faradaic reactions, such as the creation of reactive oxygen species.^30,39,40^ These potential safety concerns notwithstanding, such conducting polymers present a new avenue for OFS therapy with the potential to safely deliver stable electric fields across injured tissue.

In order to design such a system, one must first understand critical questions about how the local anatomy and device geometry affect the electric field distribution in the surrounding tissues. In addition, one must understand the charge generation mechanisms at play during such stimulation. It has been shown that, under the right conditions, the use of conducting polymers can lead to the sustained capacitive charge injection,^30^ but this must be carefully considered when designing electrodes. Indeed, these two aspects can have a direct influence on one another, as the shape and size will have a direct impact on the ability of an electrode to deliver capacitive charge, without Faradaic reactions, which is the optimal case from a biological safety standpoint. However, the effect of altering electrode geometry on the resultant electric field has received limited attention until this point.

A further consideration in the application of OFS is the safety of the tissues with regard to the injected charge and the electric field that this produces. The charge, and specifically the charge density, is a determining factor in the electrochemical safety of these devices as first described by the Shannon equation.^41^ This relates the charge injected in a single pulse to the charge density in neural tissue and provides a limit for safety of electrical stimulation for neural tissue. However, this is based purely on direct stimulation of neural tissue, without the intervening layers (dura and cerebrospinal fluid) found in the spinal cord. Others have attempted to estimate damage thresholds from current densities and shown safety thresholds of between 1 and 100 mA/cm^2^.^42,43^

In addition to the action of injected charge, the effect of the electric field has some safety considerations, particularly in relation to electroporation of cells. It has recently been shown that the electrode material has an effect on the threshold for electroporation,^40^ but it has generally been found that electroporation requires electric fields on the order of 1000 mV/mm.^44,45^ It may, therefore, be prudent to ensure any clinical application of OFS stay well below this level, setting a safety threshold in the region of 100–200 mV/mm.

**FIG. 1.**
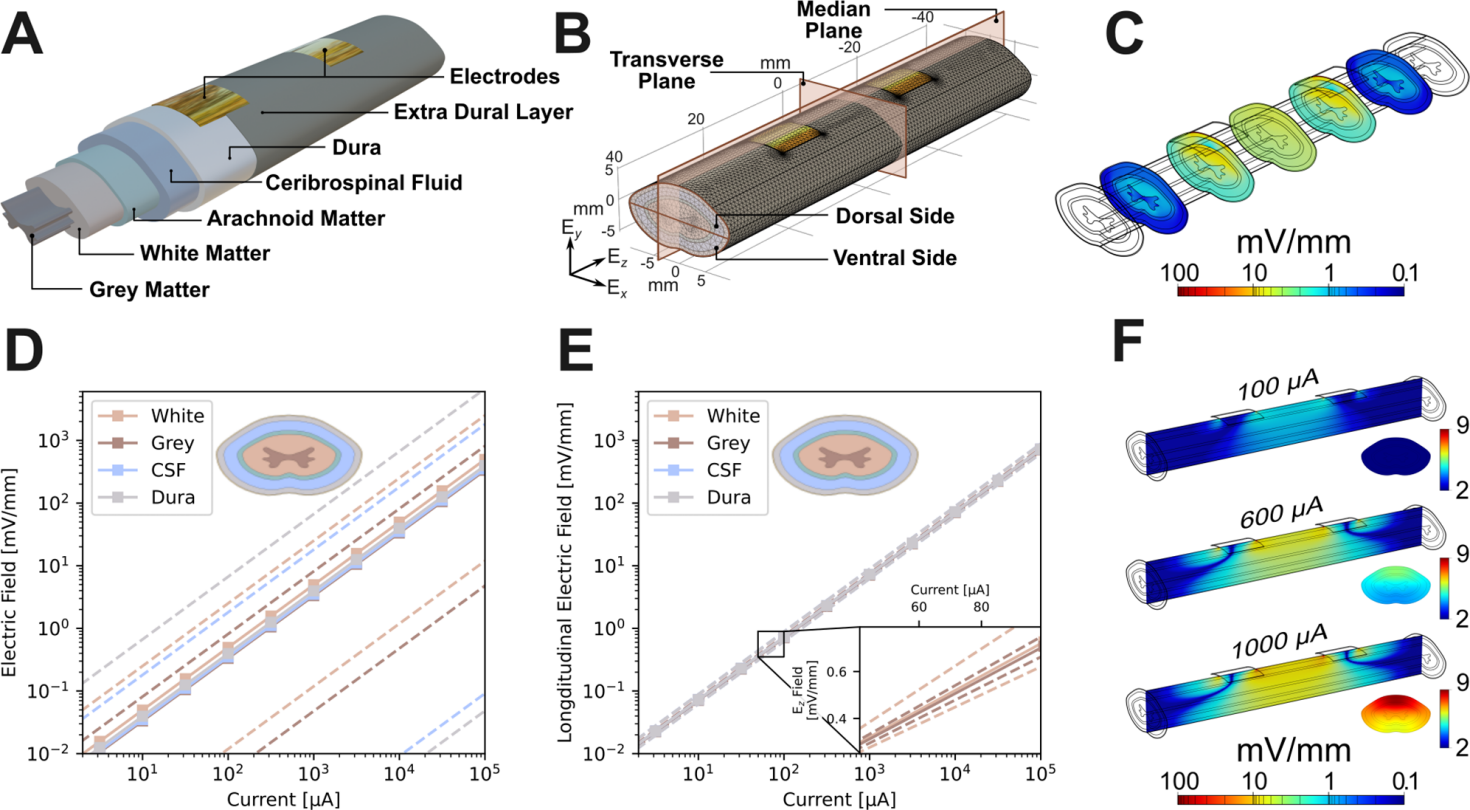
(A) Model used for FEM. (B) Finite element mesh used. The median and transverse planes used in this work are also shown. (C) Longitudinal electric field, E_*z*_, distribution inside model with 600 μA of applied direct current across the electrode pair. The electric field is shown on a log scale of color. (D) Average electric field experienced in different layers across the entire spinal cord model with increasing applied current. Dashed lines indicate maximum and minimum field in the tissue and solid lines indicate the average. (E) Longitudinal electric field measured in the plane equidistant from each electrode for different layers of the spinal cord model with increasing applied current. Dashed and solid lines have the same meaning as part (D). (F) E_*z*_ distribution for applied direct current of 100, 600, and 1000 μA.

Building on previous works, we have developed a finite element model (FEM) to study the extent of electric field penetration in a normal and pathological spinal cord of a human following epidural neurostimulation [Fig. 1(a)]. We prioritize the longitudinal component of the electric field (E_*z*_) in this study as we consider longitudinal axonal growth guidance to be a more desirable parameter during axonal pathfinding across the lesion in an attempt to reestablish synaptic connectivity. The different stages of a post-traumatic spinal cord lesion evolution during the acute stage (intramedullary spinal cord edema and hemorrhagic contusion) and subacute and chronic stages (intramedullary cyst) were modeled according to images from conventional MRI sequences of human subjects and the field penetration during these stages were analyzed. We also study the variation in the field distribution due to varying electrode geometry and placement, applied current density, and spinal cord geometry.

## II. RESULTS AND DISCUSSION

### A. Model validation

Validation of our computational model by comparison with *in vivo*, or even *in vitro*, data are challenging due to a sparsity of well-defined studies reporting such fields. While many works on DC stimulation, or low frequency OFS, report a field value, these are commonly estimated from the applied current or voltage, assuming the spinal cord is a uniform bulk conductor. However, as can be seen in our work, the different materials of the spinal cord have a pronounced effect, with the current being predominantly localized to the CSF. In addition, the vast majority of reports give a single value for the induced electric field, not considering the spatial variation between the electrodes.^46^ As far as we know, only one study has reported a directly measured and spatially defined field distribution for DC stimulation of a spinal cord.^47^ Here, the field distribution in the spinal cord of a rat was measured through differential recording of potential across two glass microelectrodes with a 1 mm separation. While the absolute values reported are likely suppressed due to the experimental limitations (particularly the saline used to keep the spinal cord hydrated), the field distribution is expected to be a good reflection of the true effects.^47^

In order to validate our model, we scaled our human spinal cord model to the dimensions of that report, adjusting the cord size, electrode geometries, and applied current to match, Fig. S2(a). Figure S2 shows comparisons of fields reported by Hurlbert and Tator,^47^ for a healthy spinal cord, and our matched model. It can be seen that field distributions in the median and transverse planes show remarkably similar patterns, limited by the resolution of the data from that report. Further we found that the magnitude of the field strengths was on a similar order when our model was adjusted to account for the difference in electrode design and the addition of saline used to keep the spinal cord hydrated. From Fig. S2, it can be seen that there remains some discrepancy between our model values and those reported by Hurlbert *et al.*; however, these can be attributed to limitations in the measurement techniques, including the granularity of the measurements, the use of glass microelectrodes, and the resection of the dura required to place the electrodes, all of which are expected to depress the recorded field potential. It is worth noting that Khan *et al.* used a measurement setup similar to that of Hurlbert *et al.* to look at the electric potential in the spinal cord of cats.^48,49^ This work showed only equipotential lines within the spinal cord, leaving the field distribution to be inferred from the distance between lines. Nonetheless, the distribution is very similar to that seen in our model, notwithstanding a modest difference in magnitude which could be explained by experimental limitations in the measurements.^48,49^ Altogether this lends confidence to the validity of our model and the applicability of the observed trends to clinical applications in humans.

Our model is also comparable to previous attempts at modeling externally generated DC electric field distribution in a human spinal cord.^50,51^ Greenebaum *et al.* reported the same charge localization within the CSF which is expected due to its high conductivity, though the use of a constant potential stimulation in that study complicates further direct comparison to our constant current simulation model. The work of Hernández-Labrado *et al.* provides a more ready comparison with our work. As with *in vivo* comparisons, the field distribution shown by Hernández-Labrado *et al.* is very similar to that seen in our model, see Fig. S3. However, the magnitude of the electric field reported by Hernández-Labrado is approximately half that seen in our model, likely due to their use of a notably larger CSF volume which would lead to more leakage current into surrounding tissues. We note that the anatomical dimensions of the spinal cord used by Hernández-Labrado *et al.* appear inconsistent with more recent human anatomy studies on which our model is based.^52,53^

A final validation of our model can be gained from considering the wider field of electrical stimulation of biological systems, and particularly other computational studies of these. From these it is well known that the electric field induced by a stimulating electrode concentrates around the edge, and particularly at corners.^45,54^ Similar concentration of the fields was found in our model as seen in Fig. S6.

Although these comparisons establish a solid foundation for understanding the field distribution produced by DC stimulations, they overlook a crucial aspect of our model. The electrodes we simulate are projected to be an integral component of a thin, flexible medical device, offering electrical insulation except for the interface between dura and electrode. This structure ensures that the current remains focused within the tissues enclosed by the dura, preventing leakage into surrounding tissues or transcellular fluids. We are confident, therefore, that our model can act as a valuable resource for guiding the development and application of the next generation of electrodes for direct current stimulation in the spinal cord.

### B. Direct current stimulation and electric field distribution

The electric field distribution generated by a constant current between two epidural electrodes placed as illustrated in Fig. 1(a) was investigated first. Figure 1(c) shows transverse slices of the longitudinal electric field, E_*z*_, distribution across the spinal cord, with an applied 600 μA current between two 10 × 10 mm electrodes. Figure 1(f) shows the electric field distribution in the median and transverse planes for three different applied currents, further shown in Fig. S3. From these, it can be seen that the highest values of E_*z*_ are situated in close proximity to the electrodes, specifically at the front edges of the two electrodes. However, the field becomes more uniform toward the transverse plane, equidistant between electrodes. This dispersion of the field away from the electrodes is a result of the prevailing current being mainly constrained within the high-conductivity CSF enveloping the white and gray matter, as depicted in Fig. S5. In this scenario, the charge carriers minimize the resistance to flow between the two electrodes by spreading throughout the CSF volume encompassing the white and gray matter. As the movement of these charge carriers (the current) induces the electric field, the broadening of current causes a homogenization of the electric field away from the electrodes. From Fig. 1(f), it can also be seen that the distribution of the field is insensitive to the applied current, but the strength of the field is strongly dependent on it. The linear dependence of field strength on applied current is further illustrated in Figs. 1(d) and 1(e). Figure 1(d) shows the average electric field strength over the entire FEM for four tissues: white matter, gray matter, CSF, and dura matter. This is calculated as a volume integral over the whole model for each tissue type. The maximum and minimum values for the field strengths are shown by dashed lines. Here, the average, maximum, and minimum field strengths are all clearly linear with applied current. Figure 1(e) shows the average E_*z*_ value in the transverse plane equidistant between the electrodes for each of these layers, where the strength of E_*z*_ is again linear with current. Here, the average is calculated as a surface integral in the transverse plane for each tissue. In this transverse plane, there is only a small variation in field strength between the dorsal and ventral sides of the model, and between different tissues of the model. This is seen in both Fig. 1(e), where there is only a small variation, on the order of 2–3 mV/mm, between maximum and minimum field values, and in Fig. 1(f), where the field strength (color) appears uniform across the transverse plane of the model, caused by the spreading out of the current, as discussed above.

### C. Effect of electrode geometry and placement on electric field distribution

**FIG. 2.**
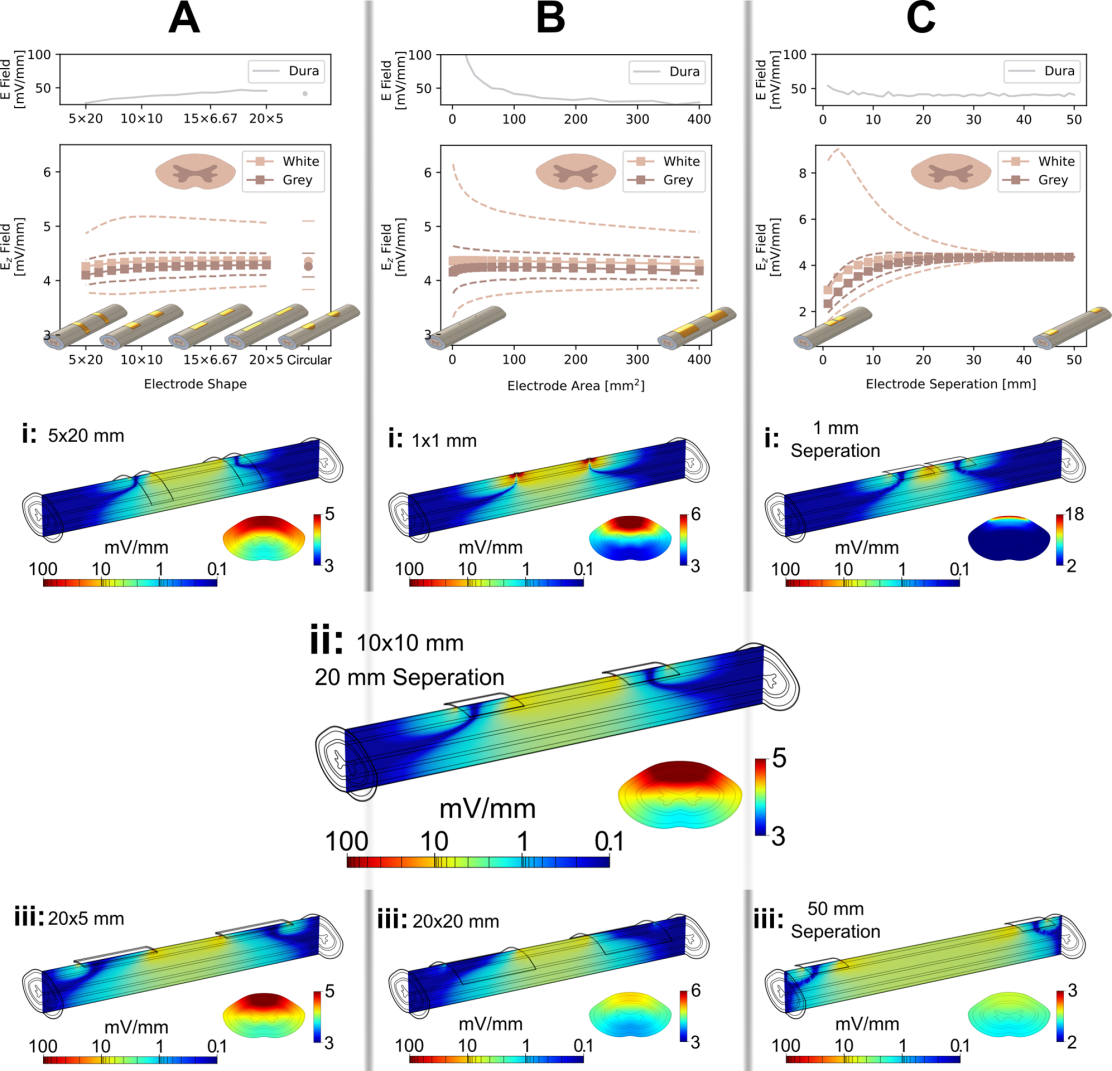
Effect of electrode shape, area, and separation on electric field distribution. (A) Average longitudinal electric field, E_*z*_, in the white and gray matter in a transverse plane equidistant from the electrodes for 100 mm^2^ area rectangular electrodes with varying aspect ratios. Dashed lines show the maximum and minimum values for these fields. A circular electrode of the same area is included for comparison. Below: E_*z*_ distribution in the median and transverse planes for three aspect ratios: (i) 5 × 20, (ii) 10 × 10, and (iii) 20 × 5 mm. (B) Average E_*z*_ in the white and gray matter for square electrodes with increasing area. Dashed lines show the maximum and minimum values for these fields. Below: E_*z*_ distribution in the median and transverse planes for two electrode areas: (i) 1 and (iii) 400 mm^2^. (C) Average E_*z*_ in the white and gray matter for 10 × 10 mm^2^ electrodes with increasing distance between electrodes. Dashed lines show the maximum and minimum values for these fields. Below: E_*z*_ distribution in the median and transverse planes for two electrode separations: (i) 1 and (iii) 50 mm. (ii) Represents an intermediary figure for parts (A)–(C), having an electrode area of 100 mm^2^ and an electrode separation of 20 mm. The geometry of (ii) is the base model throughout this work. Dashed lines in (A)–(C) show maximum and minimum fields in the tissue. The plot at the top of each part shows the maximum total electric field experienced in the dura matter.

To understand the effect of electrode geometry and placement on the induced electric field, we adjusted our model following three different parameters. In Fig. 2(a), we considered the effect of changing the shape of the contacts, while maintaining the total area and separation. In Fig. 2(b) we changed the surface area of square electrodes. In Fig. 2(c), the influence of electrode separation was investigated. All were simulated with the commonly used direct current of 600 μA,^15^ to allow ready comparison to clinical reports. Figure 2 shows, first, the maximum electric field strength in the dura matter for each parameter variation, then the average E_*z*_ value, along with the maximum and minimum values, for the white and gray matter at the transverse plane. Finally, a selection of E_*z*_ distributions along the median and transverse planes. These investigations reveal some interesting, and perhaps unexpected, results with clear clinical significance.

First, it can be seen that the average E_*z*_ in the white and gray matter at the transverse plane is almost unaffected by either electrode shape or area. This suggests that electrode dimensions could be personalized based on subject-specific considerations without detrimentally impacting the electric field distribution. However, it should be noted that the average value of E_*z*_ is not the only factor of import; the field distribution in the white and gray matter is also relevant for therapeutic applications, and the total electric field and current density in other tissues is an important safety consideration, as discussed above. The difference between the maximum and minimum E_*z*_, and the transverse field plots of Fig. 2(a) show that the shape of the electrode has only a small effect on the E_*z*_ distribution and the maximum E-field in the dura.

The area of the electrode was found to have a more pronounced effect. This was explored by varying electrode area (size) in the model from 1 to 400 mm^2^ while maintaining the same applied current [Fig. 2(b)]. As area decreases, the maximum–minimum range for the E-field in the white matter rises to nearly 3 mV/mm and the maximum E-field in the dura rises to almost 1000 mV/mm, well exceeding the safety limit of ∼100 mV/mm discussed in Sec. I. This trend in E-field distribution is clearly seen in comparison of Figs. 2(b-i) and (b-iii) wherein 1 × 1 mm electrodes lead to high fields at the electrode edge. The peak E-field values in all tissues fall rapidly as the area of the electrode is increased following an inversely proportional relationship, such that 5 × 5 mm electrodes show a maximum dura E-field value less than 10% that of a 1 × 1 mm electrodes, with only minimal further change with increasing electrode size, bringing the field strength well within the safety limit.

Of note here is the similarity between round and rectangular electrodes; it has been reported that rectangular electrodes are subject to accelerated deterioration at the corners,^55^ and it is expected that this is caused by concentration of the E-field and/or current density at these points. While this effect was small in comparison to the overall field distribution in our model, we do see some concentration of the field at the corners of the rectangular electrodes, including on the distal edge, while the circular electrode showed a broader distribution of the field along only the proximal edge, see Fig. S6. This increase in field at the corners of the rectangular electrodes has been shown to be a point of failure in stimulating electrodes^55^ and so would be best avoided in implanted devices. In addition, sharp edges and corners may cause trauma to the dura, particularly during implantation. Such trauma is known to increase the foreign body response (FBR),^56^ comprising both inflammation and fibrotic tissue creation in response to the presence of the electrode and the trauma associated with its implantation.

In addition to the FBR induced by abrasion, larger electrodes are likely to illicit a larger FBR.^57^ While simulations indicated that the severity of this foreign body reaction has little effect on the field penetration or distribution in the white and gray matter of the spinal cord some distance from the electrodes, it does lead to a large increase in the field at the electrode/tissue interface (Fig. S8), which may exceed the safety threshold set out above as the severity of the FBR is increased. This implies the use of smaller electrodes should be favored, along with minimizing abrasive corners either by using rounded electrodes or by fabricating the electrodes as part of a larger, soft, compliant thin-film electronic device.

The final geometric consideration is the distance between the electrodes, reported in Fig. 2(c). Below around 10 mm separation, the average E_*z*_ values in the white and gray matter decreases as the electrodes are placed closer together since the electric field is then unable to penetrate far into the spinal cord, exemplified in the longitudinal plot of Fig. 2(c-i). This also causes a large variation in the field distribution in the transverse plane, evidenced by the large maximum-minimum range, with the maximum value being more than double the average, and the transverse plot of Fig. 2(c-i). Above 10 mm separation, and certainly above 20 mm separation, there is little change in the field strengths or distributions. In practice, this suggests a minimum spacing of 20 mm for electrodes (or 10 mm from either side of the region of interest) be used in clinical applications in order to achieve uniform field distribution throughout the spinal cord. The importance of this separation is further considered in the presentation of the spinal cord injury itself, discussed below.

One aspect of the model not discussed extensively here is the potential difference between electrodes required to drive the 600 μA stimulation current. Our work considers only the behavior of the system after current injection from electrodes, ignoring electrode behaviors such as electrochemical effects, contact resistance, and charge depletion (although these are important design considerations as discussed in Sec. I). It was found that the potential difference across the model remained low for all simulations, below 0.5 V, as exemplified by the contact separation modeling (Fig. S7). The potential difference seen in our models suggest that, provided sufficient charge carriers are available for charge injection, the potentials required remain well within the electrochemical water window (generally −0.6 to +1.2 V for planar metal electrodes), and so irreversible Faradaic reactions would not be expected.

In summary, these findings indicate that the electrode size and shape has little impact on the longitudinal electric field. Changes in electrode area primarily affect the uniformity of the electric field rather than the magnitude of the longitudinal electric field. However, the size of an electrode should be considered carefully as high electric field strengths could be a safety concern for tissue at the electrode interface.

### D. Effect of spinal cord size and lesion on field distribution

**FIG. 3.**
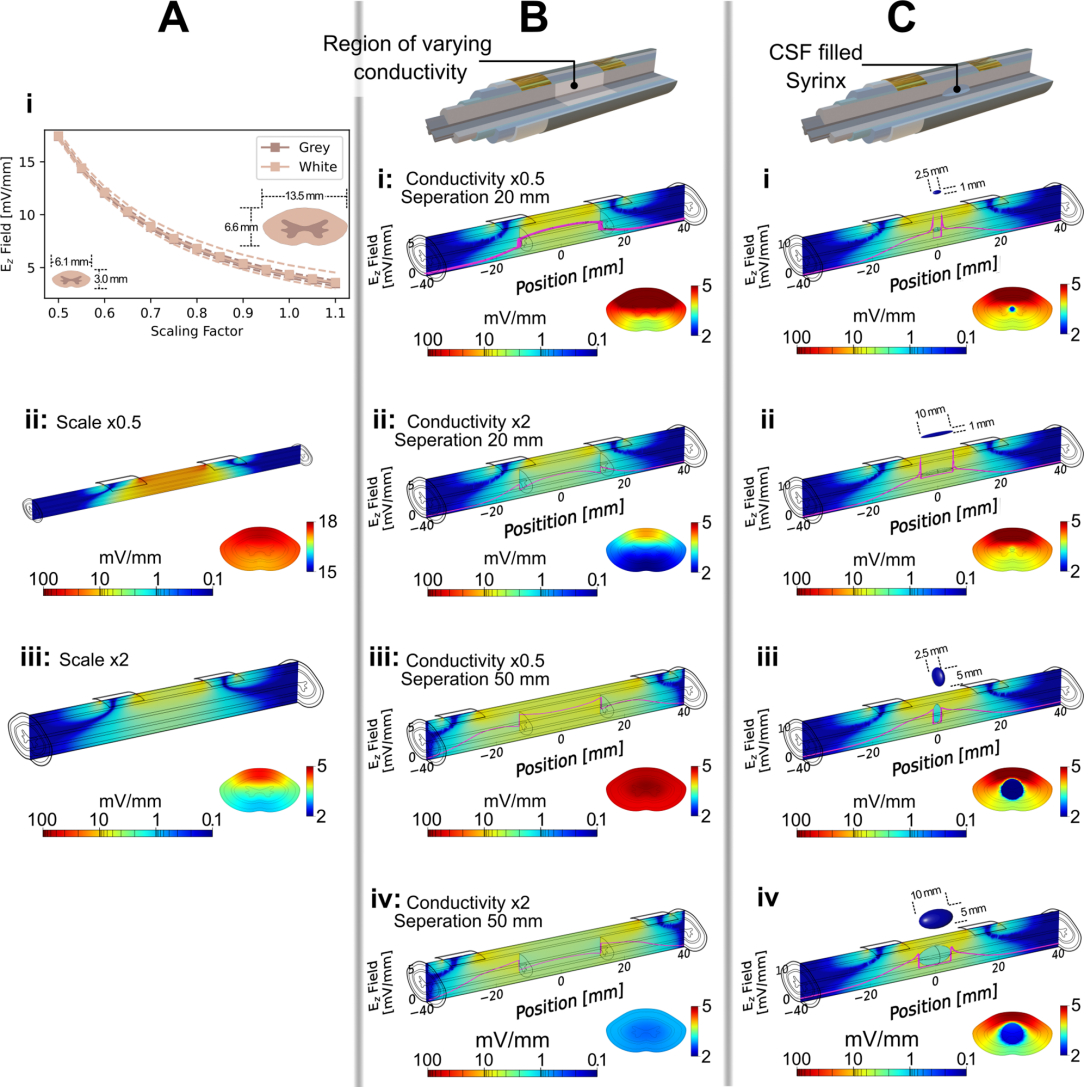
Effect of spinal cord size and lesion effects on field distribution. (A) Effect of varying the spinal cord size. (i) Average longitudinal electric field, E_*z*_, in the white and gray matter in the transverse plane equidistant from the electrodes with changing spinal cord dimensions. The original model was scaled by the factors shown. Dashed lines show the maximum and minimum values of E_*z*_ in that plane. (ii) and (iii) E_*z*_ distribution in the median and transverse planes for (ii) the smallest size, with a scaling factor of 0.5, and (iii) the largest size, with scaling factor of 2 for an applied current of 600 μA. (B) Effect of a lesion simulated as a change in conductivity of the white and gray matter in the 25 mm section highlighted in the figure. E_*z*_ distribution in the median and transverse planes, along with E_*z*_ measured through the longitudinal center of the model (pink) for (i) halved and (ii) doubled conductivities with 20 mm electrode separation, and (iii) halved and (iv) doubled conductivities with 50 mm electrode separation. (C) Effect of introducing a cyst, or syrinx, filled with CSF, of different dimensions displayed the same as part (B). Cyst dimensions are (i) 2.5 × 1, (ii) 10 × 1, (iii) 2.5 × 5, and (iv) 10 × 5 mm.

In order to understand the effect of spinal cord size on field distribution, we scaled the X and Y dimensions of our model of the white and gray matter from the original geometry, while maintaining the thickness of the other layers. A scaling range of 0.5–1.1 was used, covering spinal cord sizes from 6.1 × 3.0 mm (slightly smaller than any spinal cord level between C1 and L1) to 13.5 × 6.6 mm (slightly larger than the largest level, C5) based on conventional MRI sequences from adult human subjects.^58^ This approach accounts for the wide range of spinal cord sizes, taking into account both inter- and intra-subject variability in order to guide future device design based on the required electric field for specific areas of interest within the spinal cord. The thickness of the layers surrounding the white and gray matter were kept constant as little data are available on the variability of these values, or the correlation between changes in white/gray matter dimensions and those of surrounding layers. Figure 3(a) shows some results of this scaling. From Fig. 3(a-i), it can be seen that the average E_*z*_ value at the transverse plane of the white and gray tissues is inversely proportional to the scaling factor (and hence size). Figures 3(a-ii) and (a-iii) show the E_*z*_ field distribution for the largest and smallest of the models, respectively, for an applied current of 600 μA, from which it can be seen that similar field distributions are seen, but that the magnitude of these fields is dependent on the spinal cord size. One aspect of these findings worth noting is the relatively small variation in field strengths. Even with the inverse relationship between the model dimensions and induced field, the calculated field strengths change by only a few mV/mm. In the context of the current clinical therapeutic range (0.01–200 mV/mm), variations in spinal cord dimensions are expected to have little impact on patient outcome, at least until a much better understanding of the required field strengths is developed.

Alongside the size of the spinal cord itself, we also investigated the impact of acute, subacute, and chronic SCI on field distribution [Figs. 3(b) and 3(c)]. These injuries were modeled in two distinct ways. In Fig. 3(b), the conductivity of a 25 mm segment of the white and gray matter was either halved [Figs. 3(b-i) and 3(b-iii)] or doubled [Figs. 3(b-ii) and 3(b-iv)] from the values listed in Table I. These injury modalities can be ascribed to pathological changes that occur during the acute stages of spinal cord injury including intramedullary spinal cord edema and intraparenchymal hemorrhage. Following the findings of Sec. II C on the importance of electrode placement, Fig. 3(b) covers two electrode positions: with a 20 mm separation [Figs. 3(b-i) and 3(b-ii)], positioning the electrodes above the edges of the simulated spinal cord lesion, and with 50 mm separation [Figs. 3(b-iii) and 3(b-iv)], giving the electrodes 12.5 mm spacing between lesion boundary and electrode edge.

**TABLE I.** Electrical conductivities for biological tissues used in FEM analysis.

Biological tissue	Conductivity (S/m)	Reference
Gray matter	0.230	Geddes *et al.*^59^
White matter (longitudinal)	0.600	Ranck *et al.*^60^
White matter (transverse)	0.083	Ranck *et al.*^60^
Cerebrospinal fluid	1.700	Geddes *et al.*^59^
Dura	0.600	Lempka *et al.*^61^
Extra-dural tissue	0.250	Howell *et al.*^62^
FBR tissue	0.110	Zander *et al.*^63^
Arachnoid matter	0.125	Jiang *et al.*^64^

There are several important features to note from Fig. 3(b) relevant to clinical application. First, a reduction in conductivity leads to an enhancement in the electric field in that tissue, and vice versa. This is to be expected from electromagnetic theory but here leads to a sharp step in the field strength due to the unrealistically sharp transition between regions of higher and lower conductivity. It should be noted, however, that this is only a small change and a significant E_*z*_, well within the values reported in clinical applications, is still seen inside the simulated lesion even in the high conductivity case. If the form of damage in a patient is known, this finding can be used to guide the choice of applied current—in tissue that is higher in conductivity, a higher current may be applied to ensure the field strength in the damaged tissue is at the appropriate level, and a lower current can be used if the conductivity is reduced. Of note, the work of Hurlbert *et al.*, discussed above, showed an increase in the field strength after SCI, suggesting a fall in conductivity of the affected tissue, while Khan *et al.*, reported a fall in the field strength, suggesting the opposite for similar stage of injury.^47–49^

The other feature of interest is the marked difference in the E_*z*_ distribution in the region of interest: with a 20 mm separation, there is a clear gradation of the field from dorsal to ventral side of the spinal cord. On the other hand, the distribution is remarkably uniform for the 50 mm electrode separation. The 50 mm separation also leads to a more uniform distribution in the longitudinal direction. These features can be seen in both the transverse and median plane images of Fig. 3(b). This strongly suggests that some distance between the electrode edge and lesion boundary will be highly beneficial in therapeutic settings as a more uniform field distribution is desirable in order to deliver similar stimulation throughout the spinal cord.^36^ In Sec. II C, a spacing between electrodes of 20 mm was suggested when looking at a single plane equidistant between the two electrodes. This means a distance of 10 mm from each electrode to the plane of interest. This 10 mm figure can be expounded upon here by saying 10 mm should be the minimum spacing between an electrode and the lesion edge in order to deliver a uniform electric field over the lesion site.

In Fig. 3(c), an intramedullary cyst is introduced to the center of the model, with the cyst center at the intersection of the transverse plane and the longitudinal center of the model, to simulate post-traumatic syringomyelia seen in subacute and chronic SCI.^65^ As the cyst is known to evolve with time,^66–68^ an understanding of the electric field in its vicinity is of interest and can provide insight into some reported clinical outcomes of OFS therapy. Figures 3(c-i)–3(c-iv) show the E_*z*_ distribution in the spinal cord with four different size cysts, as shown at the top of each part. Some interesting features can be seen in these which can be attributed to the shape of the cysts and the high conductivity of the CSF they contain. In both Figs. 3(c-i) and 3(c-ii), a sharp spike in E_*z*_ is seen at either end of the cyst, caused by the concentration of charge carriers at the relatively sharp ends of the 1 mm diameter cysts as they pass into the high conductivity CSF. Conversely, the wider cysts of Figs. 3(c-iii) and 3(c-iv) show a markedly reduced spike, as there is less of a concentrating effect as the charge carriers are able to enter the CSF over a wider area.

The other feature to note is the region of low E_*z*_ inside the cyst, clearly seen in the transverse slices. This is unsurprising due to the high conductivity of the CSF and is likely a contributing factor in reports that have shown axonal regrowth around the outside of the syrinx with OFS, but little growth into the syrinx itself.^22^ It should be noted, however, that there remains some reasonable field inside the cyst, suggesting that axonal regrowth into the cyst would remain possible. This should be investigated further to develop a deeper understanding of the clinical implications for OFS in the presence of intramedullary cysts. Nonetheless, considering the limitations of surgical interventions alone,^69^ complementing decompression surgery with OFS therapy during the subacute and chronic stages of SCI holds great potential to improving patient outcomes.

## III. LIMITATIONS

A notable limitation of this work stems from the modeling of the electrodes as perfectly conformal to the dorsal surface. This is rarely the case, even with next-generation flexible devices. However, the study of FBR conductivity, in relation to the field penetration of the white and gray matter, gives some reassurance that this should be a minor concern in the case of controlled current stimulation. One possible outcome non-ideal interfacing between the electrodes and the spinal cord is current leakage to surrounding tissue, in turn reducing the current penetrating the spinal cord. This has the potential to reduce the electric field induced at the site of interest, leading to suboptimal outcomes. This should be carefully considered when designing electrodes for such applications, motivating the use of highly conformal materials, as well as potentially including an insulating region around the active electrode to minimize current leakage.

Another limitation is that this work considers only the behavior of the system after charge has been injected into the model and ignores electrochemical effects such as Faradaic reactions that may form cytotoxic byproducts. Judicious choice of electrode material is expected minimize these reactions, but that is beyond the scope of this study.

Finally, the consideration given to the various lesion types in Sec. II D should be considered a first order approximation. Lesions are never as well defined as those modeled, and so the discontinuities in electric field seen would not be expected to occur naturally. Additionally, as the fluid-filled cyst matures, it is encased in glial scar tissue, which is likely to have a further modifying effect on the penetration of the electric field.

## IV. CONCLUSION

In conclusion, a FEM model was created to understand the electric field distribution in the spinal cord created by direct current stimulation between two epidurally placed electrodes. The findings indicate that electrode shape and size have minimal impact on the field magnitude, with maximal E_*z*_ values consistently observed near the electrodes regardless of applied current. However, changes in electrode area significantly affect the uniformity of the electric field. It was found that small electrodes (<25 mm^2^) may generate dangerously high, localized electric fields at the electrode/tissue interface compared to larger electrodes operating at the same current. Exploring the effects of electrode spacing indicated a sub-20 mm electrode separation exhibits a clear gradation of E_*z*_ values from the dorsal to ventral side of the spinal cord, while 30–50 mm separations result in a more uniform distribution. The geometry of spinal cord was found to have a notable effect on the electric field strength indicating that anatomical variations should be considered when selecting the applied current. As a final consideration, the effect of a SCI on the generated electric field was considered. The results suggest that maintaining a minimum spacing of 10 mm between the contact edge and spinal cord lesion allows for a more uniform electric field distribution across the lesion. Altogether these findings emphasize the importance of considering electrode geometry and placement to safely achieve uniform electric field distribution.

## V. METHODS

A 3D model of the spinal cord was constructed using COMSOL Multiphysics computer aided design kernel, based on published anatomical parameters, Fig. 1(a). Briefly, the model consisted of white and gray matter, constructed following previous work on FEM modeling of the spinal cord^63^ and scaled to the size of the human spinal cord at the C5 level.^58^ This was surrounded by a 0.8 mm thick layer of arachnoid tissue, followed by 2 mm of cerebrospinal fluid (CSF), and a 1 mm thick layer of dura matter. Finally, the model was encased in a thin, 0.2 mm, layer of extra-dural tissue. Electrodes were placed on the extra-dural tissue with no gap between the electrode and extra-dural tissue surfaces. The extra-dural tissue in this area was converted to “foreign body reaction” tissue (FBR) to simulate the expected formation of scar tissue associated with the introduction of such a foreign body, the effect of which is discussed later. The inclusion of a thin layer of liquid between the electrode and FBR tissue was considered, but ultimately rejected because the layer would be too thin to accurately capture in the FEM and its effect could instead be captured by changing the FBR electrical properties. Furthermore, the effect of changes to the FBR electrical properties on the field distribution inside the spinal cord was found to be minimal, indicating the inclusion of another model element between the FBR and the electrode was unnecessary.

Unless otherwise stated, electrodes were 10 × 10 mm^2^, placed on the dorsal surface of the model, with a 20 mm separation between the closest edges. The influence of both the size and placement of these electrodes is investigated below.

To evaluate the distribution of the electric field throughout the model, COMSOL Multiphysics was used to solve Poisson's equation for the electric field distribution based on published electrical properties of the relevant tissues, see Table I. The finite elements of the FEM, known as the mesh, were created using the standard free tetrahedral meshing tool, with a minimum element size of 0.8 mm. The use of this mesh size was validated by performing a mesh refinement study. This showed minimal variation in results (<0.1% difference in electric field strength between the most course and most fine meshes used), while minimizing the computational load, see Fig. S1.

COMSOL models were solved in the time-independent mode to reduce computational complexity. Time independent analysis is appropriate here due to the long time scales (15 min) on which the OFS current polarity is reversed. In this case, any transient effects of changing polarity will be outweighed by the steady-state behavior of the system.

In this work, all stimulation was current controlled, with a fixed stimulation current applied and the required voltage adjusted to meet this demand. Current controlled stimulation is chosen here as it is more commonly found in clinical applications.

Previous FEM work, focused on high frequency stimulation, has shown that inclusion of the tissues described is necessary for accurate modeling of electric fields inside the spinal cord, but that additional components, such as the vertebral bone, are unnecessary.^63^ This was confirmed for our model by comparison of models with and without an additional 15 mm layer of bone. We also considered the inclusion of a CSF-filled central canal through the longitudinal center of the gray matter, in-keeping with anatomical accuracy. In these comparisons, variation of less than 1% in both the electric field and current density values in all materials was seen. Given this, the model was limited to that described above in order to reduce computational costs.

All simulations were performed in COMSOL Multiphysiscs version 5.6 using a Dell Latitude 5411 laptop (Intel^®^ Core™ i7-10850 2.70 GHz processor, 32 GB RAM).

## SUPPLEMENTARY MATERIAL

See the supplementary material for details of further COMSOL simulation results and data.

## Data Availability

The data that support the findings of this study are available within the article and its supplementary material.
